# Impact of adenotonsillectomy on pediatric quality of life: review of the literature

**DOI:** 10.1186/s13052-017-0424-2

**Published:** 2017-11-25

**Authors:** Sara Torretta, Chiara Rosazza, Maria Elisabetta Pace, Elisabetta Iofrida, Paola Marchisio

**Affiliations:** 1 Department of Clinical Sciences and Community Health, University of Milan; Otolaryngological Unit, Fondazione IRCCS Ca’ Granda Ospedale Maggiore Policlinico, Via F. Sforza 35, 20100 Milan, Italy; 2 Department of Pathophysiology and Transplantation, University of Milan; Pediatric Highly Intensive Care Unit, Fondazione IRCCS Ca’ Granda Ospedale Maggiore Policlinico, Milan, Italy

**Keywords:** Adenotonsillectomy, Children, Tonsillitis, OSAS, Quality of life

## Abstract

Adenotonsillectomy (ADT) is one of the most widely used procedures in the treatment of paediatric recurrent acute tonsillitis (RAT) and obstructive sleep apnoea syndrome (OSAS), both of which have significant repercussions on the patients’ quality of life (QoL). The purpose of our review of literature was to highlight the great variety of tools that are currently used to evaluate QoL in children, to examine data available on their efficacy and the feasibility of their use in daily clinical practice, and to determine possible limitations related to an indirect and subjective assessment of QoL in children.

Although the use of different parameters makes it difficult to compare the published studies, an analysis of the evidence currently available in the literature suggests that ADT has a generally positive impact on the QoL (especially in case of OSAS). It also highlights the importance of combining tonsillectomy and adenoidectomy in the treatment of OSAS, and documents the comparability of tonsillectomy and tonsillotomy in improving obstructive symptoms. In conclusion, our findings suggest that literature supports that ADT is associated with positive changes in QOL; however further studies using comparable standardised criteria are necessary to confirm the size and duration of this benefit.

## Background 

Adenotonsillectomy (ADT) is one of the most widely used surgical procedures in children. Its main indications are: obstructive sleep apnoea syndrome (OSAS), recurrent acute streptococcal pharyngotonsillitis, recurrent middle ear infections, hearing impairment, and, in selected cases, periodic fever with aphthous stomatitis, pharyngitis and adenopathy (PFAPA); all of these conditions have significant repercussions on the quality of life (QoL) of patients and their families [1, 2].

Many studies have assessed the efficacy of surgical treatment, specifically in reducing the number of episodes of pharyngeal pain and resolving OSAS - related respiratory disorders, and found that it has a generally positive effect on obstruction and, although to a lesser and more time-limited extent, on the number of recurrent infections [4–15].

However, the effect of ADT on QoL parameters is more uncertain because differences in the criteria of assessment make the studies difficult to compare.

The aim of this review is to asses the impact of ADT on the QoL of paediatric patients, and to highlight the great variety of tools that are currently used to evaluate QoL in children, to examine data available on their efficacy and the feasibility of their use in daily clinical practice, and to determine possible limitations related to an indirect and subjective assessment of QoL in children.

## Methods

A systematic literature review was performed using the Medline database and Cochrane Library up to January 31, 2017 on all articles concerning evaluation of quality of life in children with adenotonsillar disease. All articles published in peer - reviewed journals in English or Italian language were considered to be eligible for review. Main keywords used in the database search included “adenotonsillectomy”, “tonsillectomy”, “obstructive sleep apnoea”, “sleep disrupted breathing”, “recurrent tonsillitis”, “quality of life (QoL)”, “health-related QoL”, “validated QoL questionnaire”.

## Validated instruments for QoL and neurobehavioural assessment

Most of the studies that have specifically considered the impact of tonsillectomy and/or ADT on the QoL of children with adenotonsillar diseases have used generic QoL questionnaires, or more specific questionnaires concerning sleep disorders and pharyngeal symptoms attributable to tonsillar problems [4–15] (Table 1). The use of a validated questionnaire allows a more objective assessment of the QoL, and the evaluation of variations over time, for example in relation to treatment. It is also important to consider the improvement in terms of neurological functioning that the treatment may determine, and this can be achieved through the use of validated instruments that assess the cognitive performance of the child.

**Table 1:** Validated instruments for the evaluation of QoL in children with adenotonsillar disease

	Day-to-day life functioning	Sleep symptoms	Recurrent infections	Behaviour and social functioning	Scholastic performance and cognitive functioning	Physical functioning	Emotional functioning
Children’s Quality of Life Questionnaire (CH-QOL)	X						
PedsQL Measurement Model for Pediatric Quality of Life Inventory (PedsQL)	X				X	X	X
TNO-AZL Preschool children Quality of Life (TAPQOL)				X	X	X	X
Child Health Questionnaire (CHQ)	X			X			X
Glasgow Children’s Benefit Inventory (GCBI)				X	X	X	X
Short Form (36) Health Survey (SF-36)				X		X	X
Obstructive Sleep Apnea-18 (OSA-18)		X	X			X	X
Post Tonsillectomy QOL Questionnaire			X	X			X
14-item Paediatric Throat Disorders Outcome Test (IT-14)	X	X	X				
Brouillette questionnaire		X	X				
EuroQol Visual Analogue Scale	X	X	X	X			X
Wechsler Intelligence Scale for Children (WISC)				X	X		
Raven Progressive Matrices (RPM)					X		
School Performance Test (SPT)					X		
Kaufman Assessment Battery for Children (K-ABC)					X		

### Generic health related questionnaires

- The Children’s Quality of Life Questionnaire (CH-QOL) is a QoL tool originally designed for the use in healthy children, but later also applied to disease-related contexts; it is made up of 15 scales for a total of 55 items, exploring all the different fields of day-to-day life functioning of the child [16].
- The PedsQL Measurement Model for Pediatric Quality of Life Inventory (PedsQL) is a standardized, generic assessment instrument that provides a modular approach to measure health related QoL in healthy children and adolescents and those with acute and chronic disorders. This instrument consists of 23 items evaluating physical, emotional, social and scholastic functioning, and it integrates both generic core scales and disease-specific modules [17].
- The TNO-AZL Preschool children Quality of Life (TAPQOL) is the first multi-dimensional questionnaire evaluating health related QoL that was specifically designed for preschool children aged 1–5 years. It is a 43 items questionnaire, consisting of 12 multi-item scales that consider physical, social, cognitive, and emotional functioning; each scale contains 3 to 7 items, and the questionnaire is usually designed to refer to the preceding 3 months, although it can be adjusted to take into consideration a longer span of time. Data currently available in literature seem to suggest that this questionnaire is a valid and reliable instrument for the evaluation of parent reported health related QoL in infants and toddlers [18].
- The Child Health Questionnaires (CHQ) are a family of general pediatric QoL surveys that have been designed and normed for children aged 5 to 18 years. There are two available versions of this questionnaire, one that is designed to be directly submitted to children older than 10 years, and the parent form, in which the parent is asked to express his/her opinion on the general QoL and health related QoL of the child. The parent form is available in 2 lengths, a 28 questions version and an extended 50 questions version, and it measures 14 physical and psychosocial aspects of the everyday life of the child [19]. The accuracy of the CHQ 28 questions parent form in assessing the impact of adenotonsillar disease on children’s quality of life has been specifically evaluated in a recent British study with positive results [20].
- The Glasgow Children’s Benefit Inventory (GCBI) comprises 24 questions on the consequences of a specified intervention on various aspects of the day-to-day child life, without reference to any specific symptoms, and it can be apply to children of any age. The questionnaire was proven to be valid and reliable by different studies, and it includes 4 dimensions in the pattern of responses, relating to emotion, physical health, learning, and vitality. Although not restricted to any branch of paediatric medicine, it is eminently suitable for use in the field of paediatric otolaryngology [21].
- The Short Form (36) Health Survey (SF-36) consists in eight scaled scores, which are the weighted sums of each section. The eight sections are: vitality, physical functioning, bodily pain, general health perceptions, physical role functioning, emotional role functioning, social role functioning, mental health. Scores range between 0 and 100, with lower scores corresponding to more significant disability and higher scores to slighter disability [22, 23].

### Disease specific health related questionnaires

- The Obstructive Sleep Apnoea-18 (OSA-18) questionnaire consists in 18 questions concerning sleep disturbances, physical symptoms, emotional distress, daytime function, and caregiver concerns. Each question can be scored 1–7 and so the final score ranges from 18 to 126: a score of <60 indicates a minimal impact on the QoL; a score of 60–80 a moderate impact; and a score of >80 a highly negative impact [5, 6]. On the basis of our revision of literature, it is the most used tool for the assessment of QoL in children with adenotonsillar disease [6].
- The Post Tonsillectomy QoL Questionnaire is used to evaluate outcome measures for tonsillar surgery, specifically the frequency of tonsillitis per year, absences from work (or school), doctor visits or need for antibiotic prescriptions, and feelings of well-being (investigating self-consciousness, embarrassment, easy distraction, self-esteem, confidence and self-care, learning, concentration, liveliness, fun with friends and leisure activities, family harmony and overall satisfaction) [24].
- The 14-items Pediatric Throat Disorders Outcome Test (IT-14) was validated by the Clinical Audit and Practice Advisory Group of ENT UK in 2010 and consists in 14 questions specifically aimed at determining the impact of tonsillar disease on everyday activities. It is divided into two sections that cover two complementary and equally significant aspects of tonsillar disease: obstructive respiratory problems (five questions) and oropharyngeal infectious recurrences (nine questions) [25, 26]. Each question is scored 1–5, and the total score is directly proportional to the impact of the disease on disease-specific QoL of the patient [27].

### Symptoms questionnaires

- The Brouillette questionnaire is a symptom questionnaire that was created to decrease the need for polysomnography monitoring and to select children candidate to adenotonsillectomy. Brouillette questionnaire investigates the presence of respiratory sleep disorders and the frequency of apnoea and pathological snoring. Each item is scored from 0 to 3 (0 = absence of symptoms, 1 = occasional symptoms, 2 = frequent symptoms, and 3 = constant symptoms), and the overall score is directly proportional to disease severity [24].
- EuroQol Visual Analogue Scale is a questionnaire specifically designed to evaluate obstructive respiratory symptoms and the recurrence of tonsillar infections. The EQ-5D questionnaire is made up of two components: health status description and evaluation. In the description part, health status is measured in terms of five dimensions (5D): mobility, self-care, usual activities, pain/discomfort, and anxiety/depression. In the evaluation part, the respondents evaluate their overall health status using a visual analogue scale [28].

### Neurobehavioural comorbidities

- The Wechsler Intelligence Scale for Children (WISC) evaluates global intellectual functioning and it is composed of a Verbal Scale (testing language and expression), and a Performance Scale (testing visuo-spatial and motor skills). The Wechsler Intelligence Scale for Children (WISC) is an individually administered intelligence test for children aged 6 to 16 years. The Fifth Edition (WISC-V; Wechsler, 2014) is the most current version. It generates a Full Scale IQ (formerly known as an intelligence quotient or IQ score) that represents a child’s general intellectual ability. It also provides five primary index scores corresponding to discrete cognitive domains: Verbal Comprehension Index, Visual Spatial Index, Fluid Reasoning Index, Working Memory Index, and Processing Speed Index [29].
- The Raven Progressive Matrices (RPM) consist in a group of tests that are widely used to evaluate patients with neurological impairment and their purpose is to assess the performance in two specific cognitive fields, i.e. the educative ability (the ability to make meaning out of confusion) and the reproductive ability (the ability to absorb and subsequently reproduce an information that has been explicitly reported from one person to another). These tests consist in a series of diagrams or designs with a missing part which should be chosen by the patients among a number of available options [30].
- The School Performance Test (SPT) is a useful tool for the evaluation of cognitive abilities, and more specifically scholastic performance in older children (7 to 12 years). It is a standardized psychometric instrument composed of 3 subtests, focusing respectively on writing of dictated words, arithmetic (oral solution of problems and calculation of written arithmetical operations) and reading (recognition of words apart from the context) [31].
- The Kaufman Assessment Battery for Children (K-ABC) is a clinical instrument designed in 1983 and subsequently revised, that assesses cognitive development in children aged from 2.5 to 12.5 years. This tool is composed of 16 subtests, grouped into a mental processing set and an achievement set, which yield separate global scores; the mental processing set can be furtherly divided into a subset of tests requiring primarily sequential processing of information and a second subset requiring simultaneous processing, with separate global scores for each [32].

## The impact of adenotonsillectomy on QoL in children with OSAS

Obstructive respiratory sleep disorders can negatively affect memory processes and reduce attention levels, thus having a negative impact on learning and educational performance. The most widely accepted pathogenic mechanism is that hypoxia and hypercapnia due to obstructive apnoea may cause damage to the prefrontal cortex, thus altering the normal pattern of sleep and affecting complex cognitive functions [3]. It is well known that disrupted sleep architecture from frequent arousals during sleep has a negative effect on children daytime function too, as some studies reported that sleep disruption would be even more predictive of daytime deficits compared to hypoxia [33].

An analysis of literature shows that ADT leads to a significant short- and long-term improvement in the QoL (as demonstrated by the reduction in the total OSA-18 score), thus indicating its use in the first-line treatment of children with OSAS [4]. This improvement is not related to gender, age or the degree of obesity, but to the severity of the underlying disease: it is greater in patients with moderate or severe disease than in those with milder symptoms [5], with the most significant changes concerning organic manifestations [7]. The short-term improvement observed in patients with respiratory sleep disorders persists for at least six months [5, 7, 8], although some children have experienced the reappearance of symptoms during the follow-up. This mainly occurs in normal weighted females aged >6 years, and may be partially attributable to an incomplete therapeutic response, although some authors have hypothesised that it may also be due to a change in the perception of symptoms by the parents of pubertal children, who may overestimate QoL issues because of the profound changes their children are going through [4].

Furthermore, as symptoms in some patients persist even after surgery, a number of studies have been carried out specifically aiming at identifying risk factors for treatment failure [10, 11, 13, 14]. Among these, allergic rhinitis and turbinate hypertrophy may account for 20% of surgical failure. Allergic rhinitis should be considered a risk factor for symptomatic recurrences in OSAS patients, because it can lead to alterations in the quality of sleep, excessive daytime drowsiness, stress and depression, which may have a significant effect on post-surgical QoL [13, 14].

A few studies have investigated the effect of ADT on QoL using both objective and subjective data [9], and some of them have shown that surgery has led to significant changes in terms of polysomnographic parameters, such as a reduction in the number of apnoeic episodes and arousals, and increased oxygen saturation levels [10–12].

Kang et al. found that changes in QoL recorded by means of the OSA-18 questionnaire were not uniform, and that the improvement was more marked in the items related to sleep disturbances [9]. They also found a positive correlation between OSA-18 scores and the number of apnoeic episodes revealed by means of polysomnography in normal weighted females aged >6 years with severe adenotonsillar hypertrophy.

A few studies have compared the beneficial effects of ADT to watchful waiting. One 2014 study observed a considerable post-surgical improvement in QoL in terms both of clinical outcome and parental perception revealed by the OSA-18 score, in comparison with a not surgical group, although the possibility of a placebo effect was pointed out [15].

Mohsen et al. [24], evaluating the symptoms before and after ADT using the Brouillette score, have reported a significant post-surgical score reduction that confirms the positive effect of ADT on QoL.

The third instrument that has been widely used to evaluate the QoL of patients undergoing ADT is the Paediatric throat disorders outcome tool (T-14): various studies have found a significant reduction in the T-14 score within 2–3 months, thus suggesting that surgical treatment has immediate positive repercussions on QoL [25, 34], and prospective studies have shown that the benefit continues for 6 to 12 months [26, 34, 35]. However, after reaching its maximum during the first 6 months, the score remains substantially stable after 12 and 24 months, thus indicating that that there is no subsequent further improvement [35].

Even the use of a validated questionnaire has some limitations: it is an indirect evaluation that may be influenced by the subjective perception of the parent or guardian completing the questionnaire. It is also particularly important to take into account the confounding nature of intervention bias, i.e. parental conviction that the child must inevitably benefit of a better QoL after the operation; such bias arises from the desire of the parent to defend the appropriateness of his or her decision to allow the child to undergo surgery under general anaesthesia [34, 35].

Finally, many of the currently available consider different parameters, thus making it difficult to compare the data deriving from different studies and to formulate general conclusions concerning the impact of ADT on the overall QoL of the patient.

Concerning neurobehavioural evaluation, Ikeda et al. have investigated the intellectual capacities and school performances of children with OSAS before and one and six months after tonsillectomy or ADT, using Raven’s Coloured Progressive Matrices and the School Performance Test (SPT). They found a statistically significant improvement in intellectual performance immediately after surgery and six months later, and the children undergoing surgery obtained significantly higher scores at all three SPT tasks at both the post-operative assessments. The authors concluded that these results were attributable to the improvement in nightly rest due to the resolution of the children’s obstructive apnoea problems, which increased their ability to concentrate and to make the most of their learning potential [31].

In a more recent study, Feng et al. looked for differences in the intelligence quotients (IQs) and concentration capacities of children with OSAS before and six months after adenoidectomy or ADT. Global intellectual function was evaluated using the validated Chinese Wechsler Intelligence Scale for Children. On the basis of the scores obtained, each study participant was allocated a verbal IQ (VIQ), a performance IQ (PIQ), a global IQ (FIQ), and a concentration factor (factor C). The authors reported a significant post-surgical increase in VIQ, PIQ and FIQ values, as well as in the concentration capacity of the patients undergoing adenoidectomy alone, and the scores were even higher in those undergoing ADT. The results of this study suggest that early surgery has a positive effect on the school performance and the behavioural profile of children with OSAS, thus leading to an improvement in their overall QoL [29], and underline the importance of combining tonsillectomy and adenoidectomy in such patients.

Most of the published data concerning the impact of OSAS on neurocognitive functions come from studies conducted in school aged children and young adults, while fewer data are available regarding pre-schoolers, despite the fact that this age range has a high incidence of respiratory sleep disorders. Landau et al., however, specifically evaluated the difference between the pre- and post-operative behaviour and neurocognitive performance of ADT-treated children with a history of OSAS aged 2.5–5 years. The characteristics of their sleep, their QoL, and their neurobehavioural profiles were assessed using validated questionnaires and tests, including in particular the Kaufman Assessment Battery for Children (K-ABC), immediately before and 12 months after surgery; healthy children were used as controls. The findings showed that both internalising and externalising behavioural problems and alterations in some cognitive and executive functions could already be detected in pre-schoolers with a history of OSAS. One year after undergoing ADT, the surgically treated children had similar scores to those of the healthy controls at the tests designed to assess cognitive and executive functions and the questionnaires designed to evaluate their behaviour showed a marked reduction in behavioural disturbances and attention deficits, thus suggesting that these problems are reversible if respiratory sleep disorders are treated properly and timely [36].

### TONSILLOTOMY VS tonsillectomy in children with OSAS

Data deriving from a recent revision of literature suggest that tonsillectomy can improve sleep - related quality of life and reduce negative behaviour (such as anxiety and emotional lability) in children with OSAS undergoing surgical treatment, at least at short term follow up [37, 38].

Over recent years, there has been a clear increase in the use of tonsillotomy (the sub-total removal of tonsillar tissue, which is less invasive and allows a more rapid post-operative recovery) as an alternative to traditional tonsillectomy in the treatment of children with OSAS [39]. Some recent systematic reviews of the literature have highlighted the fact that currently available data indicate that the efficacy of the two procedures in treating obstructive apnoea is comparable: the post-operative reduction in symptom severity reported by the parents (mainly using the OSA-18 questionnaire) was the same in the two groups, as was the improvement in objective polysomnographic parameters [39]. At the same time, in comparison with tonsillectomy, tonsillotomy reduces post-procedural pain and the need for analgesic drugs, as well as the risk of post-operative bleeding [40, 41].

A recent meta-analysis of studies carried out between 1999 and 2014 investigated whether there was any difference in the impact of the two procedures on the QoL of children with OSAS. The considered studies evaluated the QoL using the Glasgow Children’s Benefit Inventory (GCBI), which was administered to the parents after their children had undergone surgery, and revealed a clear improvement in the overall QoL of the children, without any statistically significant difference between the two methods [21]. The results of a recent retrospective study in which the QoL of children who had undergone tonsillotomy with adenoidectomy or ADT was assessed using the Post-tonsillectomy Quality of Life Questionnaire were more clearly in favour of partial tonsillectomy [28].

There are no published data coming from prospective studies of post-tonsillotomy or post-tonsillectomy QoL in children, but there is a study of young adults aged 16–25 years with obstructive respiratory sleep disorders. The data were collected by means of two validated questionnaires, the Short Form (36) Health Survey (SF36) and the EuroQol Visual Analogue Scale. There was no statistically significant difference in the percentage of snoring relapses or in the rate of upper airway infections between the subjects who underwent traditional tonsillectomy and those who underwent tonsillotomy; surgery led to a clear improvement in SF-36 scores one year after partial or total tonsillectomy, with no statistically significant difference between the two procedures. The improvement in QoL was long-lasting, and was still present after six years [42].

## Impact of ADT on recurrent acute tonsillitis

The currently available evidence suggests that ADT has only a moderate effect in children with recurrent acute tonsillitis (RAT) [43–46], as it leads to a slight but significant reduction in the number of episodes of pharyngeal pain only in patients with severe symptoms [43, 44].

On the other hand, it is known that that RAT greatly limits the everyday activities of patients and their families, and that the parents of children who have undergone ADT frequently report that the operation has a generally positive effect and consequently a benefit in terms of QoL of the patient. In support of this consideration, some studies suggest that ADT in paediatric patients with RAT leads to a substantial improvement in various parameters relating to the severity of symptoms and the psychosocial sphere [35, 46], as evaluated by means of standardised questionnaires specifically concerning pharyngeal disorders such as the Pediatric Throat Disorders Outcome Measure (IT-14) [34], or more generally related to aspects of the QoL of the children and their parents (the Paediatric QoL Inventory, the Pre-School Children’s QoL Questionnaire, the Children’s QoL Questionnaire, the Child-Health Questionnaire-Parental Form) [47–49]. Consequently, some authors [46] believe that before proposing ADT, consideration should be given to qualitative parameters relating to local symptoms (pharyngeal burning, dysphagia, halitosis, cough, alterations in the tone of voice, the presence of respiratory sleep disorders, and tonsillar alterations such as hyperemia, purulent exudate, *caseum* and lymphadenopathy), systemic symptoms (fever, asthenia, general malaise, weight loss or scarce growth, nausea), and psychosocial factors relating to education, socialisation, and the familial and psychological environment, in addition to the classic criterion of the number of reported episodes of acute tonsillitis.

However, there are currently few published controlled studies designed with the aim of evaluating the impact of ADT on the QoL of patients with RAT, and they are difficult to compare because of their different outcomes and the lack of standardised parameters. Lock [47] used the Paediatric QoL Inventory to analyse the psychic and psychosocial components, and found a slight difference in favour of the surgical patients 12 months after treatment in comparison with controls. However, Van Staaij [48] found no statistically significant difference between the two groups 24 months after treatment in any of the items investigated by means of the Pre-School Children’s QoL Questionnaire (administered to children aged 2–5 years), the Children’s QoL Questionnaire (administered children aged >5 years) and the Child Health Questionnaire Parental Form (completed by the parents).

Furthermore, a recent meta-analysis [43] has reported that the only study providing data concerning the use of analgesics [47] found that there was no significant difference in the number of reported sore throat episodes between the patients who underwent ADT and the non-surgical group during the two year follow-up, although a slightly higher improvement in terms of QoL was seen in the surgical group.

Another recent review of literature focused on sleep, cognitive, behavioural, and health outcomes of tonsillectomy versus watchful waiting in children with recurrent throat infections, and it found that surgical intervention reduced the number of tonsillitis and the days of absence from school in the first year after the procedure, while it had a much less significant long-term benefit, and that there was no significant difference in QoL between the group of children who underwent tonsillectomy and the watchful waiting group [50].

Another factor contributing to the QoL is the number of lost schooldays, concerning which the literature shows that children undergoing ADT miss an average of 2.3 days less than the average of six days missed by children not undergoing surgery [43].

In conclusion, although the findings of some studies are encouraging and suggest that ADT can improve various factors relating to the psychosocial and physical condition of paediatric patients with RAT, there is still no univocal evidence of sufficient quality indicating that it is beneficial in terms of increasing the QoL of the patient. There is therefore a need for controlled randomised studies aiming at evaluating changes in terms of QoL after surgery on the basis of selected, standardised and reproducible parameters. It also follows that the parents of children with RAT who are candidates for ADT must be informed that the operation does not guarantee any certain benefit concerning the QoL of the patients or their families.

## Conclusions

Although differences in the parameters used makes it difficult to compare the various studies, the currently available evidence suggests that ADT has a positive impact on the overall QoL of paediatric patients, especially those with OSAS and respiratory sleep disorders, even if a certain number of patients may experience the recurrence of their symptoms after surgery.

The published studies show that ADT offers OSAS patients important cognitive and intellectual benefits within a short interval of time after surgical intervention, and that behavioural disturbances can be rapidly reversed if surgery is undertaken promptly. They also show the importance of combining tonsillectomy and adenoidectomy in the treatment of OSAS, and the substantially similar efficacy of tonsillectomy and tonsillotomy in improving the parameters relating to obstructive symptoms. Although some studies suggest that ADT also has a positive impact on the QoL of patients with RAT, the benefit is less marked, and there is still a need for more specific, high-quality studies using comparable and standardised parameters in order to clarify this point.

## Abbreviations

ADT: Adenotonsillectomy
FIQ: Global intelligence quotients
GCBI: Glasgow Children’s Benefit Inventory
IQs: Intelligence quotients
IT-14: Pediatric Throat Disorders Outcome Measure
OSA-18: Obstructive Sleep Apnea-18
OSAS: Obstructive sleep apnea syndrome
PIQ: Performance intelligence quotients
QoL: Quality of life
RAT: Recurrent acute tonsillitis
SF36: Short Form (36) Health Survey
SPT: School Performance Test
VIQ: Verbal intelligence quotients
